# Early and Direct Endoscopic Stone Removal in the Moderate Grade of Acute Cholangitis with Choledocholithiasis Was Safe and Effective: A Prospective Study

**DOI:** 10.3390/life12122000

**Published:** 2022-11-30

**Authors:** Chih-Ming Liang, Yi-Chun Chiu, Lung-Sheng Lu, Cheng-Kun Wu, Fai-Meng Sou, Shao-Ming Chiu, Yu-Chi Lee, Pao-Yuan Huang, Seng-Kee Chuah, Chung-Mou Kuo

**Affiliations:** 1 Division of Hepatogastroenterology, Department of Internal Medicine, Kaohsiung Chang Gung Memorial Hospital, Kaohsiung 83301, Taiwan; 2 Chang Gung University College of Medicine, Kaohsiung 83301, Taiwan

**Keywords:** cholangiopancreatography, endoscopic papillary balloon dilation, endoscopic retrograde endoscopic sphincterotomy, post-endoscopic retrograde cholangiopancreatography pancreatitis

## Abstract

Background: Evidence supporting the feasibility of single-stage stone removal in patients with a moderate grade of acute cholangitis remains insufficient. The maximal size of a common bile-duct stone suitable for removal during a single-stage ERCP in a moderate grade of acute cholangitis is unknown. Methods: We prospectively enrolled 196 endoscopic retrograde cholangiopancreatography (ERCP)-naïve patients diagnosed with acute cholangitis and choledocholithiasis. For eligible patients, single-stage treatment involved stone removal at initial ERCP. Results: A total of 123 patients were included in the final analysis. The success rate of complete stone extraction was similar between patients with mild and moderate grades of acute cholangitis (89.2% vs. 95.9%; *p* = 0. 181). Complication rates were comparable between the two groups. In the moderate grade of the cholangitis group, among patients who underwent early single-stage ERCP, the length of hospitalization declined as short as the patients in the mild grade of cholangitis (10.6 ± 6.2 vs. 10.1 ± 5.1 days; *p* = 0.408). In the multivariate analysis, early ERCP indicated shorter hospitalization times (≤10 days) (odds ratio (OR), 3.981; *p* = 0.001). A stone size less than 1.5 cm presented a high success rate (98.0%) for complete stone removal. Conclusions: Single-stage retrograde endoscopic stone removal in mild and moderate grades of acute cholangitis may be safe and effective, which can obviate the requirement for a second session, thus reducing medical expenses. ClinicalTrials.gov: NCT03754491.

## 1. Introduction

Acute cholangitis is a medical emergency. Choledocholithiasis is the most common cause of biliary obstruction leading to cholangitis [1,2]. Delayed diagnosis and treatment of patients with acute cholangitis are associated with high mortality [3,4]. Therefore, early intervention with endoscopic retrograde cholangiopancreatography (ERCP) (≤72 h following diagnosis) is recommended for patients with acute cholangitis [5]. In the 2013 Tokyo guidelines [6], patients with a mild grade of acute cholangitis may undergo common bile-duct (CBD) stone removal in the initial ERCP simultaneously. However, patients with the moderate grade of acute cholangitis with choledocholithiasis require early endoscopic or percutaneous biliary drainage (PTBD), or surgical biliary drainage with a T-tube. After the patient’s general condition improves, CBD stones can be removed. The timing of the second ERCP for stone removal in moderate cholangitis remains variable in clinical practice. Furthermore, single-stage endoscopic treatment for mild to moderate grades of acute cholangitis associated with choledocholithiasis averted the need for a second session of ERCP, thus reducing medical expenses [7,8,9,10,11]. Therefore, the 2018 Tokyo Guidelines recommend that simultaneous CBD stone treatment and biliary drainage could be performed in patients with mild to moderate grades of acute cholangitis [12]. However, evidence supporting the feasibility of single-stage stone removal in patients with the moderate grade of acute cholangitis remains insufficient. Moreover, the maximal size of a common bile stone suitable for removal during a single-stage ERCP in mild and moderate grades of acute cholangitis is unknown. Therefore, we conducted this prospective trial to evaluate the efficacy and safety of single-stage retrograde endoscopic CBD stone removal in patients with mild and moderate grades of acute cholangitis associated with choledocholithiasis.

## 2. Patients and Methods

### 2.1. Study Design

We enrolled 196 ERCP-naïve patients diagnosed with acute cholangitis and choledocholithiasis by ultrasounds or a computed tomography scan in the emergency room (ER) between November 2018 and February 2020 at Kaohsiung Chang Gung Memorial Hospital, Taiwan. As part of the single-stage treatment, we performed stone removal during the initial ERCP session. Early ERCP was defined as ERCP occurring 72 h or less following diagnosis in the ER [5].

### 2.2. Research Ethics

The institutional review board and the ethics committee of Chang Gung Memorial Hospital, Taiwan, approved this study (201701050A3), which was registered at ClinicalTrials.gov (NCT03754491). We gathered written informed consent from all included patients before the trial.

### 2.3. Inclusion Criteria

Eligible participants were at least 20 years old. The ERCP-naïve patients were diagnosed with mild and moderate acute cholangitis in the emergency room. The stage classification of acute cholangitis was based on the 2013 and 2018 Tokyo Guidelines [6,12], with a sensitivity of 91.8% and a specificity of 77.7% [13].

### 2.4. Eligibility Criteria

The study exclusion criteria included current use of antiplatelet agents (n = 3), severe acute cholangitis (n = 8), inadequate sedation (n = 14), biliary sludge (n = 26), papilla not found (n = 4), tumor-related obstruction (n = 3), and initial percutaneous biliary drainage (PTBD) (n = 15). Some patients would receive PTBD initially at the ER if the ERCP was not available on holiday.

### 2.5. ERCP Procedure

Five experienced endoscopists, who conducted an average of 100 procedures per year and had experience with >500 ERCPs, performed the procedures. A side-view endoscope (JF-260V and TJF-240, Olympus Corp., Tokyo, Japan), a cholangiographic catheter (PR-113Q, Olympus Corp., Tokyo, Japan), and a 0.035-inch Guide wire (Jagwire*™* High-Performance Guide wire; Boston Scientific, Natick, MA, USA) were used in the ERCP procedures. A balloon-tipped catheter ([length, 5.5 cm; width, 8–20 mm] Wire-guided CRE™ balloon; Boston Scientific, Natick, MA, USA) was performed to extend papilla orifice dilatation (EPBD). The balloon was inflated with saline solution to reach 8 to 20 mm diameter and to dilate the papilla with a progressive increase in pressure from 3 to 8 atmospheres for three minutes depending on CBD stone size [14]. Endoscopic sphincterotomy (EST) was performed using the standard pull-sphincterotomes (ENDO-FLEX GmbH, Voerde, Germany). In cases where biliary cannulation was difficult, we performed limited precut EST or fistulotomy (Needle Knife, pointed type, ENDO-FLEX GmbH, Voerde, Germany) or trans-pancreatic EST [15]. A pancreatic duct stent was placed for preventing post-ERCP pancreatitis (PEP) if the pancreas duct cannulation occurred twice or more. At the same time, 100 mg of indomethacin was administered anally to all patients who did not have a history of allergy [16]. Aggressive intravenous hydration (including 3 mL/kg/h during ERCP, a 20-mL/kg bolus, and 3 mL/kg/h for eight hours after ERCP) with lactated Ringer’s solution was administered to all patients without contraindications [17]. CBD stones were extracted using a balloon and/or basket catheter. A retrograde biliary drain with a plastic stent (ERBD) was inserted if CBD stone extraction could not be performed within one hour of the procedure, if the contrast medium bile flow was poor with papilla swelling after stone extraction, or if pus bile was noted. All patients underwent empiric antimicrobial treatment for acute cholangitis. All patients were asked to fast for at least 12 h after ERCP and received intravenous proton pump inhibitors (PPIs) in two doses, and then shifted to oral PPIs once daily for seven days.

### 2.6. Data Records

Before ERCP, we recorded the following demographic, laboratory, and clinical variables obtained in the ER. Additionally, endoscopic findings were recorded, including papilla type [18], juxtapapillary diverticulum, CBD stone size and number, and procedure methods used. The largest diameter of the stone, as the representative of size, was measured on the cholangiogram (Luminos dRF Max., Siemens, Germany). The difficult stone was defined as a stone size greater than 1.5 cm according to a previous study and literature review [19,20].

### 2.7. Statistical Analysis

Descriptive statistics, including distribution, absolute frequency, relative frequency, medians with range, and means ± standard deviation (SDs) were calculated depending on the variable type. Between-group differences for quantitative variables with normal distribution were compared using Student’s *t*-test. The differences between categorical data proportions were evaluated with a Fisher’s exact test when there were fewer than five expected cases; otherwise, we used the chi-square test. We included factors with probability (*p*) values < 0.1 in the univariate analysis in the logistic regression analysis [21]. A multivariate logistic regression model was adopted to identify independent factors of procedural success and major adverse events. A *p*-value < 0.05 was considered to indicate statistical significance in all analyses. All analyses were performed using the Statistical Package for Social Sciences (IBM SPSS^®^, version 22.0 for Windows, IBM Corp., Armonk, New York, NY, USA).

### 2.8. Endpoints

Primary outcomes were as follows: (1) success rate of complete bile-duct stone removal, (2) significant complications, including post-ERCP pancreatitis (amylase levels higher than three times the upper reference limit accompanied by abdominal pain) [22], perforation, and bleeding. Secondary outcomes included the development of pneumonia within 30 days, mortality within 30 days after ERCP, and the length and cost of hospitalization. The definition of bleeding degree for patients who did not require transfusion was “mild bleeding degree.” Cases requiring up to four units of blood were defined as “moderate bleeding degree,” and those requiring five or more units of blood for transfusion, surgery, or angiography were defined as “severe bleeding degree” [23]. Operation time during ERCP was defined as the period ranging from the beginning of cannulation to complete stone removal.

## 3. Results

### 3.1. Population Characteristics

**Figure 1:**
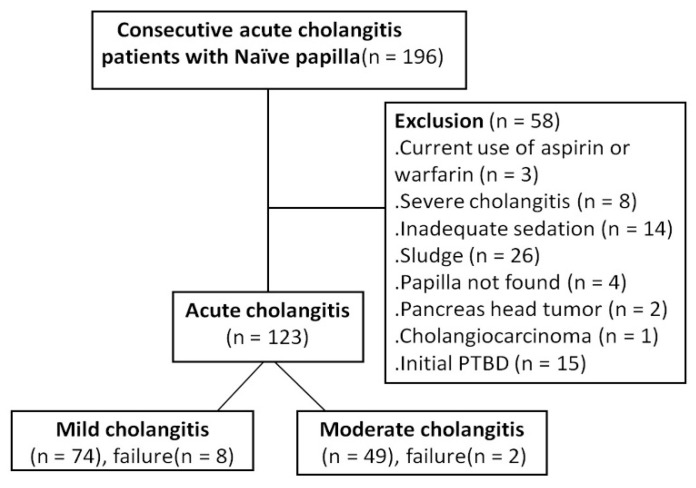
The flow diagram of the prospective enrollment of consecutive patients with mild/moderate acute cholangitis.

**Figure 2:**
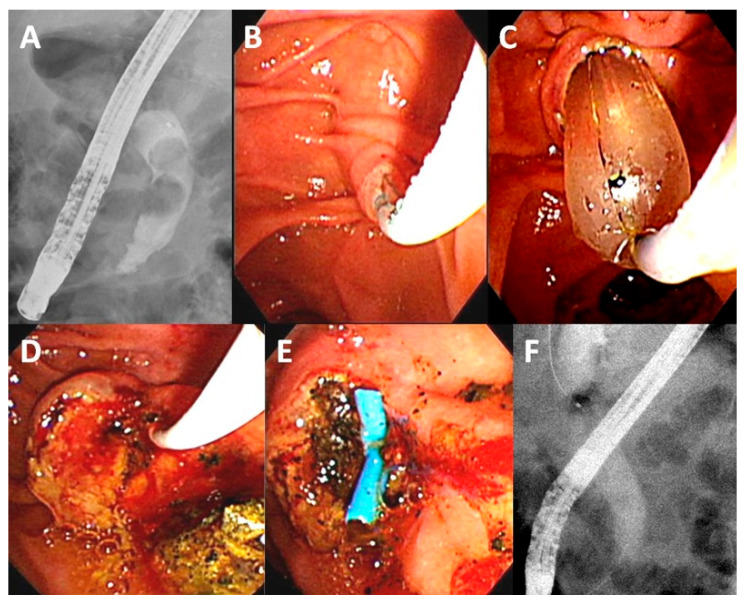
Management of choledocholithiasis in a 91-year-old male patient with moderate acute cholangitis (Body temperature: 38.2 °C, white blood count: 20.6 × 10^3^/μL, total bilirubin: 5.1 mg/dL, albumin: 3.0 mg/dL). (**A**) Cholangiogram showing two common bile-duct stones (1.6 cm, and 1.3 cm); (**B**) Perform a trans-biliary sphincterotomy with limited cutting over the papilla; (**C**) Combination with balloon dilation (15 mm); (**D**) Stones were extracted by balloon; (**E**) Placement of a plastic stent over edematous papilla for bile drainage; (**F**) Final cholangiogram showing complete stones clearance.

The study enrolled 123 patients who fulfilled the inclusion criteria, including 74 and 49 patients in the mild and moderate grades of acute cholangitis groups, respectively (Figure 1). Figure 2 presented the management of choledocholithiasis in a 91-year-old male patient with moderate acute cholangitis. There were no differences in sex, personal habits (i.e., alcohol use and smoking), white and platelet blood counts, levels of alanine transaminase, albumin, bilirubin, and alkaline phosphatase; estimated glomerular filtration rate (eGFR) between the two groups. Meanwhile, age, comorbidity rate (i.e., diabetes and hypertension), ASA score, initial body temperature (°C) in the ER, PT, APTT, and CRP were higher in the moderate cholangitis group than in the mild cholangitis group (Table 1).

### 3.2. Endoscopic Findings

There were no differences in papilla type, rates of endoscopic papillary balloon dilation (EPBD), and EST between the two groups. Stone size (0.9 ± 0.4 vs. 1.1 ± 0.5 cm; *p* = 0.157), number of stones (1.8 ± 1.2 vs. 1.4 ± 1.00; *p* = 0.078), mean CBD diameter (1.3 ± 0.4 vs. 1.5 ± 0.4 cm; *p* = 0.856), frequency of endoscopic retrograde biliary drainage (ERBD) use (21.6% vs. 30.6%; *p* = 0.261), and procedure time (24.3 ± 11.4 vs. 23.8 ± 11.4 min; *p* = 0.661) did not differ in the mild and moderate cholangitis groups. The ratio of the juxtapapillary diverticulum was higher in the moderate cholangitis group than in the mild group (55.1% vs. 36.5%; *p* = 0.042). The complete stone extraction success rate was similar in the mild and moderate grades of acute cholangitis groups (89.2% vs. 95.9%; *p* = 0.181) (Table 2).

### 3.3. Treatment Outcomes and Complications

The complication rates were comparable between the two groups (PEP, 5.4% vs. 2.0%, *p* = 0.355; bleeding, 1.4% vs. 0%, *p* = 0.414; perforation, 1.4% vs. 0%, *p* = 0.414; pneumonia, 1.4% vs. 2.0%, *p* = 0.767). However, the moderate acute cholangitis group had higher costs (4011.7 ± 3048.8 vs. 3005.7 ± 1302.7 USD; *p* < 0.001) and experienced comparable hospitalization duration (12.6 ± 9.3 vs. 11.8 ± 6.2 days; *p* = 0.326) compared to the mild grade group, as shown in Table 3. There was no case of mortality in either group. One patient experienced coffee-ground vomiting and a mild degree of bleeding after EPBD. Five patients with a mild degree of PEP were treated with conservative care, and the symptoms spontaneously resolved.

### 3.4. Timing of Single-Stage Stone Extraction during ERCP

In the subgroup analysis of early-stage ERCPs (≤72 h) (mean ± SD, 35.2 ± 20.4 h), there were similar rates of successful complete stone extraction and complications in the two groups. The hospitalization lengths (10.6 ± 6.2 vs. 10.1± 5.1 days; *p* = 0.408) and costs (2806.5 ± 1313.4 vs. 3076.5 ± 1953.9 USD; *p* = 0.312) were similar in the mild and moderate grade groups (Table 4).

The subgroup analysis of delayed ERCPs (>72 h) (mean ± SD, 161.2 ± 85.1 h) indicated similar success rates of complete stone extraction and ERCP complications in the two groups. The costs were higher (4037.0 ± 3810.0 vs. 3175.7 ± 1185.5 USD; *p* = 0.050) in the moderate grade group than in the mild group. The hospital stays were comparable in the two groups (16.4 ± 12.9 vs. 15.5 ± 6.9 days; *p* = 0.196).

**Figure 3:**
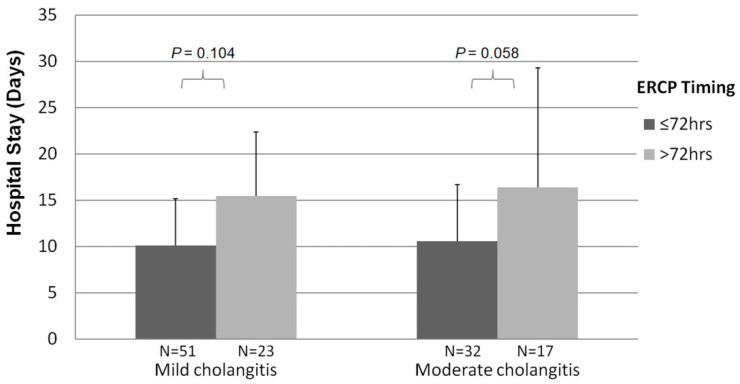
Relationship between the length of hospitalization and single-stage ERCP time from the emergent room.

Figure 3 showed the slightly longer hospital stays in the delayed ERCPs compared to the early ones but did not show a significant change for analysis in the mild acute cholangitis (*p* = 0.104) and moderate groups (*p* = 0.058).

In the univariate and multivariate analyses of the factors associated with shorter hospital stay (≤10 days), early-stage ERCP (≤72 h) indicated reduced hospitalization (odds ratio [OR], 3.981, 95% confidence interval [CI], 1.753–9.040; *p* = 0.001). Meanwhile, the presence of liver cirrhosis (OR, 0.109, 95% CI, 0.012–0.965; *p* = 0.046) was a factor predicting longer hospitalization (Table S1).

### 3.5. Stone Size during Single-Stage Stone Extraction by ERCP

Failure to remove stones occurred in 10 cases, including five cases that experienced difficult cannulation even with precut EST or fistulotomy use and five cases with simple failure of stone extraction. In the univariate and multivariate analyses of the factors associated with CBD stone extraction failure, after excluding cases of cannulation failure (n = 5), stone size greater than 1.5 cm was an independent factor prompting CBD stone extraction failure (OR, 9.263, 95% CI: 1.315–65.226; *p* = 0.025) (Table S2).

**Figure 4:**
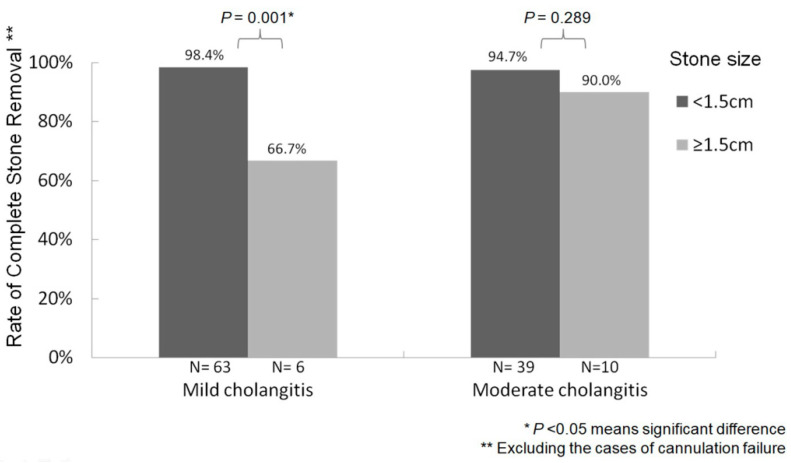
Relationship between bile-duct stone size and stone removal success rate after excluding cannulation failure cases.

The rate of complete CBD stone removal varied according to CBD stone size (i.e., <1.5 cm or ≥1.5 cm); after excluding cases with cannulation failure, higher success rates occurred among patients with CBD stones < 1.5 cm (98.4% and 94.7%) compared to those with stones ≥ 1.5 cm (66.7% and 90.0%) regardless of whether ERCP was performed in mild or moderate acute cholangitis (*p* = 0.001 and *p* = 0.289, respectively) (Figure 4).

## 4. Discussion

The most worrying issue under review in this investigation was whether direct stone extraction in mild to moderate acute cholangitis would increase complications, such as bleeding and pancreatitis. The institutional review board and ethics committee recommended we adopt high-quality prevention strategies to reduce adverse effects of ERCP, such as guidewire-based selective cannulation, long-term dilatation (at least 3 min) during EPBD [24], pancreatic duct stenting, indomethacin use, aggressive hydration [22], and PPI administration. However, adding EST may introduce a higher risk of unforeseen complications, such as bleeding (4–14.5%) [25,26]. Acute cholangitis seems to be an independent risk factor for post-EST bleeding in previous reports [16,27]. Therefore, biliary drainage without sphincterotomy is recommended in patients with severe acute cholangitis [25]. In a national, population-based study by Hung et al. [28], EPBD was the preferred method to decrease the risk of post-ERCP hemorrhage, especially in patients with liver cirrhosis or impaired renal function. Thus, we typically opted for EPBD (~90%) for stone removal in cases of acute cholangitis to reduce bleeding events. In this study, there was no case of post-EST bleeding (0/21; 0%), not even among the six cases with trans-biliary EST, ten cases with fistulotomy, three cases with limited precut EST, and two cases with trans-pancreatic EST. Only one patient experienced a mild degree of bleeding after EPBD. Therefore, the bleeding risk of EST in mild and moderate grades of acute cholangitis may be minimal and acceptable. Similarly, Ito et al. [7] reported that there were no complications, such as pancreatitis, bleeding, or perforation, with procedures performed by experienced specialists concerning immediate EST for acute suppurative cholangitis. In the previous single-stage treatment ERCP studies, Eto et al. [8] reported a 90% success rate for stone removal in patients with mild to moderate acute cholangitis, with an acceptable bleeding rate of 4% (2/50). Hung et al. [10] also reported a similar success rate and minimal bleeding rate of (0–2.2%). In our study cohort, there was also an acceptable PEP complication rate (5/123; 4.1%) after adopting strict prevention strategies.

In clinical practice, the optimal timing for stone removal in moderate acute cholangitis remains inconsistent. For example, Hui et al. [29] performed a second ERCP procedure on all patients four to eight weeks after the first session of ERCP for bile-duct stone removal. In the study by Ito et al. [7], all patients underwent a second elective EST procedure for bile-duct stone removal one week later after the first ERCP. In our study, among patients with acute cholangitis who were treated with early (≤72 h) and single-stage ERCP, the hospitalization length of the patients with the moderate grade of cholangitis declined as short as the mild grade group (10.6 ± 6.2 vs. 10.1± 5.1 days; *p* = 0.408) (Table 4). In the multivariate analysis, early single-stage ERCP (≤72 h) was an independent factor predicting shorter hospitalization (OR, 3.981; *p* = 0.001). Initially, we doubted that the patients in this study with moderate acute cholangitis who received early or delayed ERCP had different inflammation severity levels at baseline. In the sub-analysis of baseline characteristics of patients with moderate acute cholangitis who underwent early and delayed ERCP, there was no difference between the two groups in terms of age, renal function, albumin, WBC count, liver function, or bilirubin. Conversely, a higher level of CRP was noted in patients with moderate acute cholangitis who underwent early ERCP relative to delayed ERCP (123.3 ± 70.0 vs. 74.3 ± 73.3 U/L; *p* = 0.007). Therefore, it appears reasonable to suggest that the optimal timing of single-stage stone removal in the moderate cholangitis is within 72 h. Similar to studies covering the standard management of acute cholecystitis, adopting an early approach to laparoscopic cholecystectomy within 72 h of symptom onset reduces operative time, decreases the length of hospital stay and mortality, and was associated with fewer adverse postoperative outcomes [30,31,32]. The hypothesis for these likely relates to the management of foreign-body infection [33]: Removing debris from the site of injury reduces the *bacterial load* and thereby facilitates infection control. Bactibilia (the presence of bacteria in the biliary tract) increases in the presence of biliary obstruction, mainly stone obstruction, such as foreign bodies in the biliary tree [34]. The most common bacteria linked to ascending cholangitis are *Escherichia coli*, *Klebsiella*, *Enterobacter*, and *Enterococcus*, which could form the biofilm covering the surfaces of stones. This biofilm protects the bacteria from antibacterial agents and phagocytic leukocytes [35]. Therefore, prompt removal of infected stones as soon as possible in cases of acute cholangitis and cholecystitis is preferable.

In acute cholangitis, the Tokyo Guidelines 2018 (TG18) recommend 4–7 days of antibiotic treatment for patients with acute cholangitis once the source of infection is controlled, but evidence for this was graded as low (evidence level C) [36]. Most studies discussed the early ERCP for acute cholangitis regarding biliary drainage only [37]. Rare studies reported the duration of antibiotics after single-stage ERCP stone removal.

The stone size during single-stage removal in acute cholangitis is an important contributing factor to the technical difficulty of CBD stone clearance [38]. We determined that stones with a size less than 1.5 cm were not difficult to be removed and achieved higher success rates for stone removal (94.7–98.4%) than stones measuring larger than 1.5 cm (66.7–90.0%) when biliary cannulation was successful (Figure 4). Thus, trying to directly remove stones in patients with mild and moderate grades of acute cholangitis if the stone size < 1.5 cm seems to be feasible.

The limitations of the current study need to be acknowledged. First, this study was initially designed as a randomized controlled trial to compare single-stage and two-stage ERCP-based stone removal (biliary drainage first and bile stone removal one week later) in patients with moderate acute cholangitis. However, most patients refused to undergo two-stage ERCP because of the need for more than one session. Therefore, we altered the trial design to a prospective trial of single-stage ERCP in consecutive patients with mild and moderate grades of acute cholangitis. Second, a single-stage treatment for large stones (≥1.5 cm) in patients with moderate acute cholangitis might be more complicated. More research is required to assess the benefits and risks. Definitive treatment with the removal of large stones is still recommended only after the patient’s general condition becomes stable as per the recommendations of established guidelines [12].

## 5. Conclusions

Patients with moderate acute cholangitis have more comorbidities and a more severe degree of inflammation, resulting in longer hospitalization stays and higher costs than those with mild acute cholangitis. Single-stage retrograde endoscopic CBD stone removal in mild and moderate grades of acute cholangitis with choledocholithiasis may be safe and effective, especially if the stone is less than 1.5 cm, which can obviate the requirement for a second ERCP session, thus reducing medical expenses. In this study, we demonstrated the benefit of early (≤72 h) and single-stage ERCP on shorter hospital stays and reduced costs for patients with mild and moderate grades of acute cholangitis with respect to late ERCP. However, large-scale, randomized clinical trials are necessary to clarify the safety and efficacy of the single-stage approach, especially when dealing with CBD stones larger than 1.5 cm.

## Figures and Tables

**Table 1:** Demographic data for the two groups (mild and moderate acute cholangitis).

Characteristics	Mild Acute Cholangitis n = 74 (*)	Moderate Acute Cholangitis n = 49 (*)	*p*-Value
Age (year)	62.9 ± 16.1	76.0 ± 11.2	0.003
Gender (F)	33 (44.6)	18 (36.7)	0.386
Smoking	15 (20.3)	9 (18.4)	0.794
Alcohol	13 (17.6)	8 (16.3)	0.858
Diabetes Mellitus	14 (18.9)	21 (42.9)	0.004
Hypertension	37 (50.0)	35 (71.4)	0.018
ASA score I/II/II/IV	31/31/8/4	9/21/12/7	0.012
Body temperature (°C)	36.8 ± 0.9	37.8 ± 1.4	<0.001
WBC (×1000/μL)	10.0 ± 5.4	13.7 ± 4.3	0.141
Platelet (×1000/μL)	235.5 ± 91.7	194.6 ± 74.2	0.102
PT (s)	10.6 ± 0.8	11.7 ± 2.8	0.007
APTT (s)	27.4 ± 3.4	29.8 ± 4.9	0.038
Albumin (mg/dL)	3.7 ± 0.4	3.3 ± 0.5	0.488
eGFR (mL/min/1.73^2^)	70.5 ± 34.7	48.8 ± 25.0	0.063
AST (U/L)	200.8 ± 252.2	228.4 ± 192.4	0.914
ALT (U/L)	260.7 ± 209.6	243.9 ± 210.7	0.583
Bilirubin (total) (mg/dL)	5.7 ± 4.6	5.9 ± 4.4	0.265
ALK-P (U/L)	205.1 ± 133.4	231.8 ± 126.3	0.845
CRP (U/L)	45.7 ± 67.9	106.7 ± 74.0	0.017

Abbreviations: ASA, American Society of Anesthesiology score; WBC, white blood cell; PT, prothrombin time; APTT, activated partial thromboplastin time; eGFR, estimated glomerular filtration rate; AST, aspartate aminotransferase; ALT, alanine aminotransferase; ALK-P, Alkaline phosphatase; CRP, C-reactive protein; * Values are expressed as numbers and percentages in parentheses or mean ± standard deviation.

**Table 2:** Endoscopic findings and outcomes in patients with mild and moderate acute cholangitis.

Characteristics	Mild Acute Cholangitis n = 74 (*)	Moderate Acute Cholangitis n = 49 (*)	*p*-Value
Pancreatic duct filling	2 (2.8)	3 (6.1)	0.373
Papilla type(I~IV)	59/7/5/3	38/7/2/2	0.803
Periampullary diverticulum	27 (36.5)	27 (55.1)	0.042
EPBD	68 (91.9)	44 (89.8)	0.690
EST	16 (21.6)	6 (12.2)	0.184
Stones size (cm)	0.9 ± 0.4	1.1 ± 0.5	0.157
Stone number	1.8 ± 1.2	1.4 ± 1.0	0.078
Mean CBD diameter (cm)	1.3 ± 0.4	1.5 ± 0.4	0.856
Balloon extraction	65 (94.2)	45 (91.8)	0.614
Basket use	8 (11.6)	8 (16.3)	0.459
ERBD	16 (21.6)	15 (30.6)	0.261
Procedure Time (min)	24.3 ± 11.4	23.8 ± 11.4	0.661
Complete stone removal	66 (89.2)	47 (95.9)	0.181

Abbreviations: EPBD, endoscopic papillary balloon dilation; EST, endoscopic sphincterotomy; CBD, common bile duct; ERBD, Endoscopic retrograde biliary drainage; * Values are expressed as numbers and percentages in parentheses or mean ± standard deviation.

**Table 3:** Outcome and adverse events in patients with mild and moderate acute cholangitis.

Adverse Events	Mild Acute Cholangitis n = 74 (*)	Moderate Acute Cholangitis n = 49 (*)	*p*-Value
Hospital stay (days)	11.8 ± 6.2	12.6 ± 9.3	0.326
Hospital cost (USD)	2912.1 ± 1280.7	3409.8 ± 2741.9	0.026
PEP	4 (5.4)	1 (2.0)	0.355
Bleeding	1 (1.4)	0 (0)	0.414
Perforation	1 (1.4)	0 (0)	0.414
Pneumonia (30 days)	1 (1.4)	1 (2.0)	0.767
Mortality (30 days)	0 (0)	0 (0)	-

Abbreviations: PEP, post-ERCP pancreatitis. * Values are expressed as numbers and percentages in parentheses or mean ± standard deviation.

**Table 4:** Clinical characteristics and outcomes according to ERCP timing according to the type of cholangitis (mild vs. moderate).

ERCP Timing From ER (Mean ± SD) (h)	Early (≤72 h) (35.2 ± 20.4), n = 83			Delayed (>72 h) (161.2 ± 85.1), n = 40		
	Mild Cholangitis n = 51 (*)	Moderate Cholangitis n = 32 (*)	*p*-Value	Mild Cholangitis n = 23 (*)	Moderate Cholangitis n = 17 (*)	*p*-Value
Age (y/o)	62.0 ± 16.6	76.0 ± 10.6	0.004	65.0 ± 15.2	76.0 ± 12.8	0.279
CRP (U/L)	44.8 ± 63.0	123.3 ± 70.0	0.001	47.6 ± 79.0	74.3 ± 73.3	0.293
Stone ≥ 1.5 cm	2 (3.9)	7 (21.2)	0.010	5 (21.7)	3 (17.6)	0.749
Complete CBD removal	48 (94.1)	30 (93.8)	0.945	18 (78.3)	17 (100.0)	0.040
PEP	3 (5.9)	1 (3.1)	0.568	1 (4.3)	0 (0)	0.384
Bleeding	1 (2.0)	0 (0)	0.425	0 (0)	0 (0)	-
Perforation	0 (0)	0 (0)	-	1 (4.3)	0 (0)	0.384
ERBD	13 (25.5)	14 (43.8)	0.084	3 (13.0)	1 (5.9)	0.455
Hospital stay (days)	10.1 ± 5.1	10.6 ± 6.2	0.408	15.5 ±.6.9	16.4 ± 12.9	0.196
Hospital cost (USD)	2806.5 ± 1313.4	3076.5 ± 1953.9	0.312	3175.7 ± 1185.5	4037.0 ± 3810.0	0.050

Abbreviations: ERCP, endoscopic retrograde cholangiopancreatography; CRP, C-reactive protein; CBD, common bile duct; PEP, post-ERCP pancreatitis; ERBD, Endoscopic retrograde biliary drainage. * Values are expressed as numbers and percentages in parentheses or mean ± standard deviation.

## Data Availability

Not applicable.

## Supplementary Materials

The following supporting information can be downloaded at: https://www.mdpi.com/article/10.3390/life12122000/s1. Table S1: Univariate and multivariate analyses of the factors associated with reduced length of hospitalization (≤10 days); Table S2: Univariate and multivariate analyses of the factors associated with failure of the CBD stone extraction (excluding cases with cannulation failure).
