# Supplementary material for: Early and Direct Endoscopic Stone Removal in the Moderate Grade of Acute Cholangitis with Choledocholithiasis Was Safe and Effective: A Prospective Study
Source: Life (Basel). 2022 Nov 30;12(12):2000. doi: 10.3390/life12122000 (PMC9781833; doi:10.3390/life12122000)
Supplement: Supplementary file 1 [file life-12-02000-s001.zip › life-2025655-supplementary.pdf]

Table S1. Univariate and multivariate analyses of the factors associated with reduced length of hospitalization (≤10 days).

Variants	Hospital Stay (≤ 10 days) n = 68 (%)	Hospital Stay (>10 days) n = 55 (%)	Univariate OR (95% C.I.)	*p*-Value	Multivariate OR (95% C.I.)	*p*-Value
**Clinical factors**						
Age ≥ 75 y/o	29(42.6)	28(50.9)	0.907(0.339–2.426)	0.846		
Male gender	41(60.3)	31(56.4)	0.707(0.300–1.667)	0.428		
BMI ≥ 24	30(57.7)	27(60.0)	0.755(0.287–1.984)	0.569		
Diabetes	16(23.5)	19(34.5)	1.659(0.499–5.513)	0.409		
Hypertension	36(52.9)	36(65.5)	1.739(0.605–5.002)	0.305		
Cerebrovascular accident	3(4.4)	3(5.5)	0.428(0.058–3.140)	0.404		

Coronary artery disease	11(16.2)	14(25.5)	1.105(0.327–3.734)	0.872		
Liver cirrhosis	1(1.5)	7(12.7)	9.521(0.969–93.526)	0.053	0.109 (0.012–0.965)	0.046
Chronic kidney disease (eGFR < 60 mL/min/1.73^2)	5(7.4)	12(21.8)	1.940(0.411–9.170)	0.403		
Moderate acute cholangitis	27(39.7)	22(40.0)	1.668(0.637–4.367)	0.298		
**Laboratory factors**						
WBC ≥ 12,000 or < 4000 (×10^3/μL)	30(44.1)	28(50.9)	0.789(0.320–1.947)	0.607		
CRP ≥ 100 (U/I)	19(28.4)	17(31.5)	0.572(0.222–1.469)	0.245		
Bilirubin ≥ 5 (mg/dL)	30(44.1)	24(44.4)	0.768(0.322–1.832)	0.551		

**Endoscopic factors**						
Stone ≥ 1.5 cm	8(11.8)	9(16.4)	0.785(0.235–2.624)	0.695		
ERBD	15(22.1)	16(29.1)	0.452(0.179–1.141)	0.093		
ERCP timing ≤ 72 h	55(80.9)	28(50.9)	5.287(2.060–13.569)	<0.001	3.981 (1.753–9.040)	0.001

Abbreviations: OR, odds ratio; BMI, body mass index; WBC, white blood count; CRP, C-reactive protein; ERBD, Endoscopic retrograde biliary drainage; ERCP, endoscopic retrograde cholangiopancreatography.

Table S2. Univariate and multivariate analyses of the factors associated with failure of the CBD stone extraction (excluding cases with cannulation failure).

**Variants**		**Success Rate n (%)**	**Univariate OR (95% C.I.)**	***p*-Value**	**Multivariate OR (95% C.I.)**	***p*-Value**
Age (y/o)	≥ 75	50/54 (92.6)	5.040(0.546–46.522)	0.154		
	<75	63/64(98.4)				
Stage of acute cholangitis	Moderate	47/49(95.9)	0.936(0.150–5.824)	0.944		
	Mild	66/69(89.2)				
CRP	≥100	32/35(91.4)	3.703(0.591–23.217)	0.162		
	<100	79/81(97.5)				
WBC ≥ 12,000 or < 4000 (×1000/μL)	(+)	55/57(96.5)	0.703(0.113–4.369)	0.705		
	(-)	58/61(95.1)				

ERCP timing	>72 h	35/37(94.6)	0.673(0.108–4.209)	0.672		
	≤72 h	78/81(96.3)				
Diverticulum	(+)	50/51(98.0)	0.315(0.034–2.908)	0.308		
	(-)	63/67(94.0)				
Stone (cm)	≥1.5	13/16(81.3)	11.538(1.760–75.632)	0.011	9.263 (1.315–65.226)	0.025
	<1.5	100/102(98.0)				
Distal CBD	(+)	25/27(92.6)	2.347(0.371–14.828)	0.352		
narrowing	(-)	88/91(96.7)				
EPBD	(+)	104/108(96.3)	0.346(0.035–3.434)	0.364		
	(-)	9/10(90.0)				
EPT	(+)	18/21(85.7)	7.917(1.234–50.793)	0.029	6.167(0.874–43.503)	0.068

(-)	95/97(97.9)

Abbreviations: CBD, common bile duct; OR, odd, ratio; CRP, C-reactive protein; WBC, white blood count; ERCP, endoscopic retrograde cholangiopancreatography; EPBD, endoscopic papillary balloon dilation; EST, endoscopic sphincterotomy.
